# Validation of automated complex head and neck treatment planning with pencil beam scanning proton therapy

**DOI:** 10.1002/acm2.13510

**Published:** 2021-12-22

**Authors:** Samantha Grace Hedrick, Scott Petro, Alex Ward, Bart Morris

**Affiliations:** ^1^ Provision CARES Proton Therapy Center Knoxville Tennessee USA

**Keywords:** automated planning, H&N, PBS, proton therapy, scripting

## Abstract

**Background:**

Pencil beam scanning (PBS) proton therapy offers dosimetric advantages for several treatment sites, including head and neck (H&N). However, to achieve the optimal target coverage and robustness, these plans can be complex and time consuming to develop and optimize. Automating the treatment planning process can ensure a high‐quality and standardized plan, reduce burden to the planner, and decrease time‐to‐treatment. We utilized in‐house scripting to automate a four‐field multi‐field optimization (MFO) H&N planning technique.

**Methods and materials:**

Ten bilateral H&N patients were planned in RayStation v6 with a four‐field modified‐X beam configuration using MFO planning. Automation included creation of avoidance structures to control spot placement and development of standardized beams, PBS spot settings, robust optimization objectives, and patient‐specific predicted planning constraints. Each patient was planned both with and without automation to evaluate differences in planning time, perceived effort and plan quality, plan robustness, and OAR sparing.

**Results:**

On average, scripted plans required 3.2 h, compared to 4.3 h without the script. There was no difference in target coverage or plan robustness with or without automation. Automation significantly reduced mean dose to the oral cavity, parotids, esophagus, trachea, and larynx. Perceived effort was scaled from 1 (minimum effort) to 100 (maximum effort), and automation reduced perceived effort by 42% (*p* < 0.05). Two non‐scripted plans required re‐planning due to errors.

**Conclusions:**

Automation of this multi‐beam, the MFO proton planning process reduced planning time and improved OAR sparing compared to the same planning process without scripting. Scripting generation of complex structures and planning objectives reduced burden on the planner. With most current treatment planning software, this automation is simple to implement and can standardize quality of care across all treatment planners.

## 1 INTRODUCTION

Proton therapy utilizing pencil beam scanning (PBS) has been shown to improve sparing in head and neck (H&N) treatments compared to intensity modulated radiation therapy (IMRT),^1^, ^2^, ^3^ particularly in structures like the oral cavity. PBS proton therapy is rapidly advancing, with new planning and delivery techniques that could further improve sparing compared to IMRT. One such advancement is the use of clinical target volume (CTV)‐based robust optimization, which can potentially reduce the size of the dose cloud in the treatment, without sacrificing nominal coverage or robustness. In fact, studies have shown that PBS robust CTV‐based optimization, compared to planning target volume based planning, improves plan quality and robustness.^4^ With the ability to optimize robustly comes the ability to develop multi‐field optimization (MFO) plans that potentially provide better conformality and organ‐at‐risk (OAR) sparing. MFO planning has been shown to improve plan quality for H&N patients compared to single‐field optimized (SFO) planning.^5^, ^6^, ^7^ Another advancement is the widespread adoption of Monte Carlo (MC)‐based dose calculation and optimization, through the development of faster, more efficient algorithms and improved hardware. Specifically for H&N treatments, due to heterogeneities and potentially large air gaps between the patient and range shifters, MC dose calculations have been shown to be more accurate than pencil beam algorithms.^8^, ^9^, ^10^ When developing H&N planning techniques, there are several complexities to consider, including setup uncertainty, dental metal avoidance, shoulder avoidance, and heterogeneities. Increasing the number of beams can reduce the impact of each of these uncertainties, and studies have shown that increasing the number of beams in a plan can improve robustness.^11^, ^12^ Combining these planning techniques; treating with multiple beams, using robust optimization, and optimizing and calculating with MC, can clearly be beneficial for the patient. However, it can be challenging and time consuming for the planning team. These types of plans are complex, with many planning structures and techniques to utilize, and a good plan could be dependent on an experienced proton planner.^13^, ^14^ Additionally, there is the time required for calculation of the necessary perturbations during MC optimization and dose calculation. Studies have shown that reducing time‐to‐treatment, particularly for H&N patients, can have a significant impact on their survival.^15^ Based on these factors, it is important to not only treat H&N patients with the highest quality PBS proton treatment, but also do it as quickly and efficiently as possible to reduce time‐to‐treatment.

The treatment planning system used in this study is RayStation V6 (RaySearch Laboratories AB, Stockholm, Sweden). RayStation allows for user scripting with IronPython 2.7.1. The code accesses a wide range of functions within the treatment planning system beyond simple button clicks. Our clinic has been utilizing scripting for over 6 years and daily utilizes over 50 scripts, written in‐house. These scripts vary from simple scripts, such as contouring the air in a user‐selected list of contours for overrides, to complex scripts, such as importing and analyzing on‐treatment CTs or performing robust perturbation analysis. Another highly effective use of scripting is plan automation. Scripted plan generation provides several benefits to workflows, including reduced user button clicks and intervention, standardized beam arrangements and settings, and standardized robust optimization. Another advantage of scripting is standardized optimization objectives for target coverage and patient‐specific customized optimization objectives for OAR sparing. Our clinic utilizes an in‐house dose–volume prediction script based on proximity of OARs to the target. Using multiple treatment sites, such as H&N, brain, and prostate, we found the typical dose fall‐off around a target of our system. Our script then uses this fall‐off to simulate isodose rings around the target at every 10% of prescription dose. It then uses the overlap of these simulated isodose rings with OARs to predict dose volume histogram (DVH) points and mean dose. In the case of H&N, it can accurately predict mean dose for the parotids, oral cavity, larynx, and trachea, which are typically peripheral to the target. It is not used for structures like the esophagus, which is typically surrounded by the target and not well predicted. Currently, the script cannot predict SIB dose distributions, but that work is in progress. A more complete description of the OAR prediction script can be found in the supporting information.

We have written a script to automatically produce a complex MFO PBS proton therapy plan with robust beam angles and spot positions that can be utilized by planners of any proton planning experience level. It combines existing tools we have developed, including a validated H&N planning technique and predictive OAR sparing. The purpose of this study was to validate this automation in the clinic, which could potentially reduce planning and evaluation time and effort and improve plan quality. While similar tools exist for photon treatment planning,^16^, ^17^ there are few tools available for PBS proton therapy.

## 2 METHODS

### 2.1 Plan design

In this study, we chose to plan and evaluate the initial 50.0 Cobalt Gray Equivalent (CGE) phase for sequentially planned bilateral H&N patients. H&N patients at our clinic tend to have similar CTV50 target contours, including nodal volumes. Additionally, this initial phase typically requires the most planning time. Therefore, it was advantageous to standardize this phase of planning. While our clinic does utilize scripting to improve efficiency in boost planning, sequential boost plans for H&N tend to be less standardized than the initial phase, with smaller and typically more ipsilateral target volumes. Because the focus of this work was automating and standardizing complex planning processes, boost evaluations were not included in this study. However, OAR planning constraints for this initial phase did consider the necessary additional dose that would be delivered in boost phases.

**FIGURE 1:**
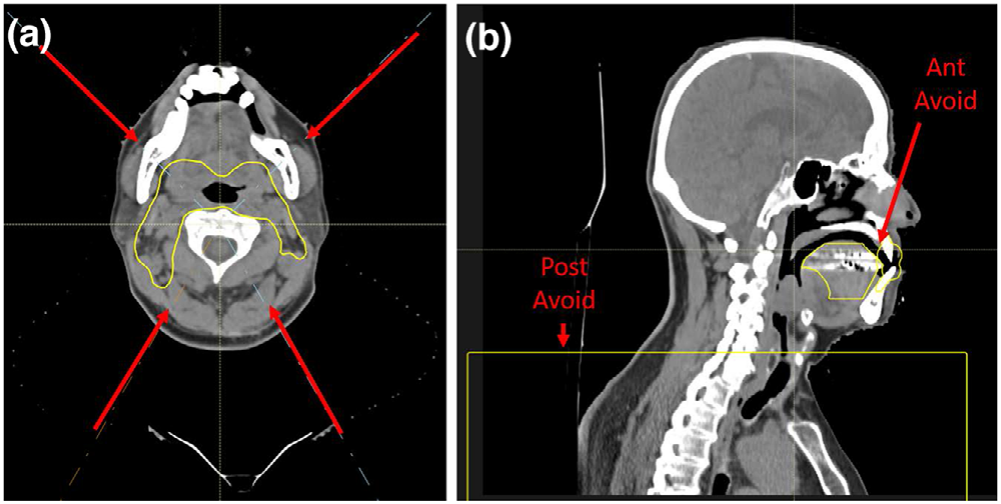
(a) Example of the four‐field modified X beam configuration, with beam angles indicated with red arrows. (b) Example of the custom avoid structures created for each patient. “Post_Avoid” is applied to the posterior beams to avoid treating through the shoulders. The “Ant_Avoid” structure is the oral cavity, minus the target, plus any dental hardware plus an 8 mm expansion. The avoid is applied to the anterior beams to avoid beams treating through metal and to reduce oral cavity dose

The standard plan design utilizes a four‐field modified X beam configuration, shown in Figure 1a, with beams at 225°, 330°, 30°, and 135°. This beam configuration was previously validated in‐house for many bilateral H&N patients and found to be consistently robust and provide the best coverage and sparing for all patients. For unilateral H&N patients, beams are chosen specifically for the patient and their target size and location. However, for bilateral H&N patients, this standard beam configuration works well for the initial 50 CGE phase due to the consistent target size and location for nearly all patients. For the sequential boost phases, custom beam angles are chosen based on the boost target size and location, so automation is not utilized in those phases. To optimize sparing and reduce uncertainties, avoidance structures were developed to limit the regions of spot placement for each beam. In RayStation, avoidance structures prevent the optimizer from placing a spot through or within the specified contour. For the anterior beams, an avoidance structure (“ant_avoid”) was created by unioning the oral cavity and any dental hardware plus an 8 mm expansion, shown in Figure 1b. This avoidance structure serves two purposes. First, it improves oral cavity sparing by allowing the posterior beams to treat the target in this region and not pass anterior beams unnecessarily through the oral cavity. Second, it reduces uncertainties caused by dental hardware. Therapists align daily to vertebra and skull. Even with a thermoplastic mask on the BoS frame (QFix, Avondale, PA), the angle of the chin can vary slightly. The daily position of dental hardware could change, affecting the range of the beam. The 8 mm margin is half of our spot size at full‐width‐half‐maximum (FWHM), so the margin allows the optimizer to place a spot near the metal, but not within a 50% dose fall‐off of the metal.

In our experience, shoulder positioning can be variable, even with shoulder pulls utilized in CT simulation. To avoid this uncertainty, posterior beams do not place spots inferior to the top of the shoulders (post_avoid), shown in Figure 1b. In the nodal region below the shoulders, only the anterior beams may treat. To further reduce uncertainty and improve robustness, an additional structure is created to control the contribution of dose from each anterior beam in the supraclavicular target area. In this region, the plan is designed to be SFO, with equal dose from each anterior beam and minimal gradients.

To reduce dose to the posterior neck and minimize dose between bilateral volumes, a midline optimization structure (PostNeck) is created by adding a margin laterally and posteriorly from the spinal cord.

The beam settings were optimized to improve conformality and reduce large deviations between minimum and maximum spot monitor unit (MU) values. Spot spacing is reduced from the default value, overlapping at the 80% dose fall‐off, to improve spot weight homogeneity and reduce biological uncertainties. The maximum MU is no greater than 150 times the minimum spot weight. The lateral target margin is reduced to improve OAR sparing and conformality. A minimum spot depth of 0.5 cm is applied to improve skin sparing. Our proton delivery system can deliver a minimum energy of 97.5 MeV, treating to a depth of 7.5 cm. For shallow targets, such as H&N, a 7.5 cm water equivalent thickness (WET) range shifter is applied to each beam. The air gap for the anterior beams is 5 cm from the most proximal point of the patient external, correlating to approximately 10 cm air gap at isocenter. For the posterior beams, a 10 cm air gap from the most proximal point of the patient external is applied, correlating to an approximate 15 cm air gap at isocenter. The larger posterior air gap is to allow for gantry‐housing clearance of our 6 degree‐of‐freedom (DOF) couch.

Automated and standard optimization objectives included CTV coverage and dose fall‐off. The CTV objectives included a minimum dose at prescription, and a robust minimum DVH of *D*97% > 97%. Dose fall‐off, an objective unique to RayStation, was utilized for the patient external contour to control conformality. A maximum dose per anterior beam is added to create the SFO region below the shoulders. A maximum dose objective is placed on a 5 mm skin rind to reduce skin dose. A maximum dose objective is placed on the posterior neck structure to reduce posterior dose. Maximum dose objectives are added to the brachial plexus, spinal cord, and brainstem.

Predicted mean doses were included for the parotids, oral cavity, larynx, and trachea using the prediction script included in the automation. Table S1 provides a list of the planning objectives.

Maximum dose objectives are added robustly and non‐robustly to the patient external to control hotspots both on the nominal plan and perturbations. As mentioned previously, an MFO plan will typically improve OAR sparing compared to an SFO plan, but the improvement comes at a cost. MFO plans are typically less inherently robust due to large gradients in the individual beam distribution that can be sensitive to daily set up changes or changes in the anatomy. To smooth gradients and improve robustness, robust optimization can be performed. For each iteration, the optimizer will consider a perturbation that not only shifts the entire patient by a user‐defined magnitude in any direction, but also shifts each beam independently from the other beams by the same magnitude. RayStation uses a minimax optimization technique to ensure optimization functions are robust for the worst‐case scenario. Our current version of RayStation only allows the user to choose independent beam optimization to move each beam in all directions. If we were to optimize in all directions, with four independent beams, our number of perturbations per iteration would be 7 203. Even with no anterior/posterior shifts included in the robust optimization, but including independent beams, the value is 1 875 perturbations per iteration. Because one of the primary goals of this study is to reduce planning time, the number of necessary perturbations per iteration was reduced as low as possible while maintaining consistent robustness across most H&N patients. When developing this H&N planning technique, we previously tested several combinations of robust optimization settings. We started with the same values used for evaluation, using a patient shift of 3 mm in all directions, with independent beams and 3.5% range uncertainty. The optimization time was prohibitively long. We then tested less robust optimization settings, removing certain directions, removing independent beam optimization, and reducing range uncertainty. Because the beams are primarily in the anterior–posterior direction, robustly optimizing in those directions does not improve robustness, it simply adds time. Additionally, robustly optimizing to 3.5% range uncertainty is not necessary, since it may artificially create a larger prescription dose cloud than necessary. In most cases, we have found that we can reduce range uncertainty optimization to 1.0% and maintain robust coverage when evaluated at 3.5%. Finally, it was found that increasing the number of treatment beams reduces the individual beam gradients. By using four beams, instead of the more common three‐beam plans for H&N proton therapy, we are able to plan without independent beams optimization. Robustness optimization settings used in this study included 0.2 cm shifts right, left, superior, and inferior and 1% range uncertainty. We found that this combination provides a clinically acceptable plan that maintains robust CTV coverage under perturbation evaluation and requires the minimal amount of optimization time, reducing the number of perturbations per iteration to only 15. Each plan was optimized with MC (5 000 ions/spot) and a final dose calculation was performed with MC (0.5% uncertainty).

### 2.2 Automation

The script provides a graphic user interface, allowing the user to choose the target CTV and the planning CT, then input the dose and number of fractions. The script will then generate the necessary planning structures, including a skin rind, “PostNeck”, and the “ant_avoid” structure. It will then pause to let the user identify the most superior point of the shoulders with the crosshairs, which indicates the top of the “post_avoid” structure. This structure is then automatically created, along with the SFO nodal region structure. The script will next create the necessary contours for OAR sparing prediction, then create a plan with the standard beam angles, beam settings, robustness settings, planning objectives, clinical goals, and patient‐specific predicted OAR objectives. In our version of RayStation, adding avoidance structures to the beam settings cannot be scripted, so the script ends before optimization, with a reminder to the user to add the avoidance structure to each beam.

### 2.3 Validation

Ten bilateral H&N patients were retrospectively planned, optimizing to 50.0 CGE in 25 fractions to the CTV50. The summary of patient characteristics is in Table 1. The boost volumes and plans were not included in this study. The CTV50 included the gross tumor volume and bilateral elective nodal volume, with coverage of pterygoids to skull base. Ten planners participated in this study. Each planner developed a plan with the script for a designated patient and without the script for a different patient. The planners were not able to compare their current plan to a previously developed plan or the approved and treated plan. Those plans created without the scripts were also not prompted to use the prediction script, only told to plan as they normally would. Beam angles, robust optimization settings, beam settings, and avoidance structures were identical for each plan, regardless of the script. These settings were chosen to be consistent with and without the script because they had been previously validated. However, in the case of non‐scripted plans, these settings had to be applied manually. The patient included the CT and structure set, with contoured CTV50 and OARs. When planning without automation, planners were given instructions on how to create the necessary avoidance structures and the list of beams and settings to be applied in optimization. For all patients, with and without the script, planners were instructed to continue optimization until they felt the plan had reached the quality that would pass both physics evaluation and physician approval. Each planner was asked to track the time and number of optimization iterations required to complete a plan. After the plan was finished, the planner completed a survey documenting time and iterations required, perceived level of plan quality, perceived effort required to make the plan, and perception of time required to complete the plan.^18^ Perceived effort was scaled from 1 to 100, where 1 was described as minimum effort and 100 was described as maximum effort. This metric was collected to determine the planner's perception of the effort required to develop a high‐quality plan and was subjective to the planner.

**TABLE 1:** Summary of patient and tumor characteristics

**Patient**	**Primary tumor location**	**Stage**	**TNM**	**CTV50 (cm^3^)**
A	Tonsil	I	T2N1M0	408.8
B	Glottis	II	T1NOM0	521.7
C	Nasopharynx	II	T1N2M0	396.3
D	Oropharynx	I	T1N1M0	438.2
E	Base of Tongue	II	T3N0M0	352.3
F	Base of Tongue	I	T2N1M0	421.9
G	Larynx	IV	T2N2M0	358.3
H	Base of Tongue	I	T1N1M0	537.0
I	Oropharynx	IV	T4N2M0	379.8
J	Oropharynx	IV	T0N2M0	381.8

## 3 RESULTS

**FIGURE 2:**
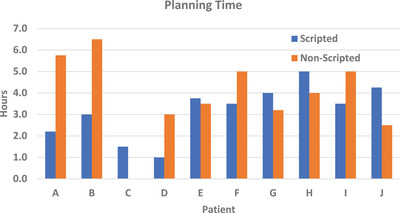
Planning time comparison, in hours, per patient. Scripted plans (blue) vs. non‐scripted plans (orange). The non‐scripted planning time for patient C is an outlier and was omitted

A paired *t*‐test statistical analysis was performed for each evaluation metric. When comparing the planning time required for scripted plans (3.2 h) and non‐scripted plans (4.3 h), there was no statistical difference. The comparison for each patient is shown in Figure 2. The planning time for patient C without the script was considered to be an outlier, at 27 h, and was not included in the average planning time value. The number of iterations required for a scripted plan was, on average, 360 compared to 430 for non‐scripted plans.

Target coverage was similar between the two types of plans. Each plan, both scripted and non‐scripted, met or exceeded the clinical target coverage goal of CTV50 *V*95% > 99%. The average scripted CTV50 *V*95% was 99.2% compared to 99.1% without the script.

**FIGURE 3:**
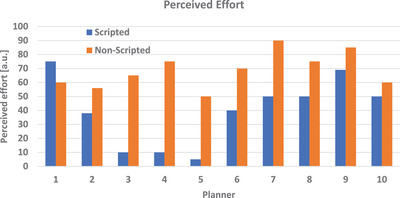
Comparison of perceived effort per planner. Perceived effort was scaled from 1‐minimal effort to 100‐maximum effort and was subjective to each planner. Scripted plans (blue) vs. non‐scripted plans (orange)

Nominal plan quality was evaluated with OAR sparing. The mean dose to the left parotid, right parotid, oral cavity, esophagus, larynx, and trachea were reported, as shown in Table 2. For each reported OAR, the script produced plans with significantly reduced mean doses compared to non‐scripted. The maximum dose to the brainstem was higher, on average, in the scripted plans (21.57 CGE) than in the non‐scripted plans (14.66 CGE). Similarly, the maximum dose to the spinal cord was higher, on average, in the scripted plans (26.94 CGE) than in the non‐scripted plans (19.95 CGE). A DVH of a single patient in shown in Figure 3, demonstrating the similarity in target coverage and differences in OAR sparing between the scripted plan (solid) and the non‐scripted plan (dashed). In this study, we chose to evaluate only the initial phase, based on the potential gains in standardization and automation. The OAR doses achieved in the manual plans are similar to the treated plans for these patients and were clinically acceptable, considering the addition of two boosts following the initial phase. Though scripting was not utilized on the boost phases, for completeness, the composite OAR doses have been included in Table S2. The boost plans were 2 phases of 10 CGE in 5 fractions each for a total of a 60 CGE and 70 CGE prescribed to their respective CTVs. The composite plans are reported using the clinically treated boost plans summed with the scripted and non‐scripted initial phase plans. The ranges of mean and maximum doses are large for this dataset due to differences in boost volumes and laterality.

**TABLE 2:** Summary of organ‐at‐risk (OAR) mean doses for scripted vs. non‐scripted plans

Structure	Scripted (CGE)	Non‐scripted (CGE)	*p*‐Value
Parotid_L	22.85	27.21	<0.05
Parotid_R	18.31	23.64	<0.05
OralCavity	26.09	29.48	<0.05
Esophagus	9.53	11.49	<0.05
Larynx	28.87	35.23	<0.05
Trachea	16.78	25.75	<0.05

Abbreviation: CGE, Cobalt Gray Equivalent.

Perceived plan quality was scaled from 1 to 100, with 1 being the lowest quality and 100 being the highest quality of the treatment plan. The average perceived plan quality was nearly identical for a scripted plan (87.8) versus non‐scripted (84.9).

**FIGURE 4:**
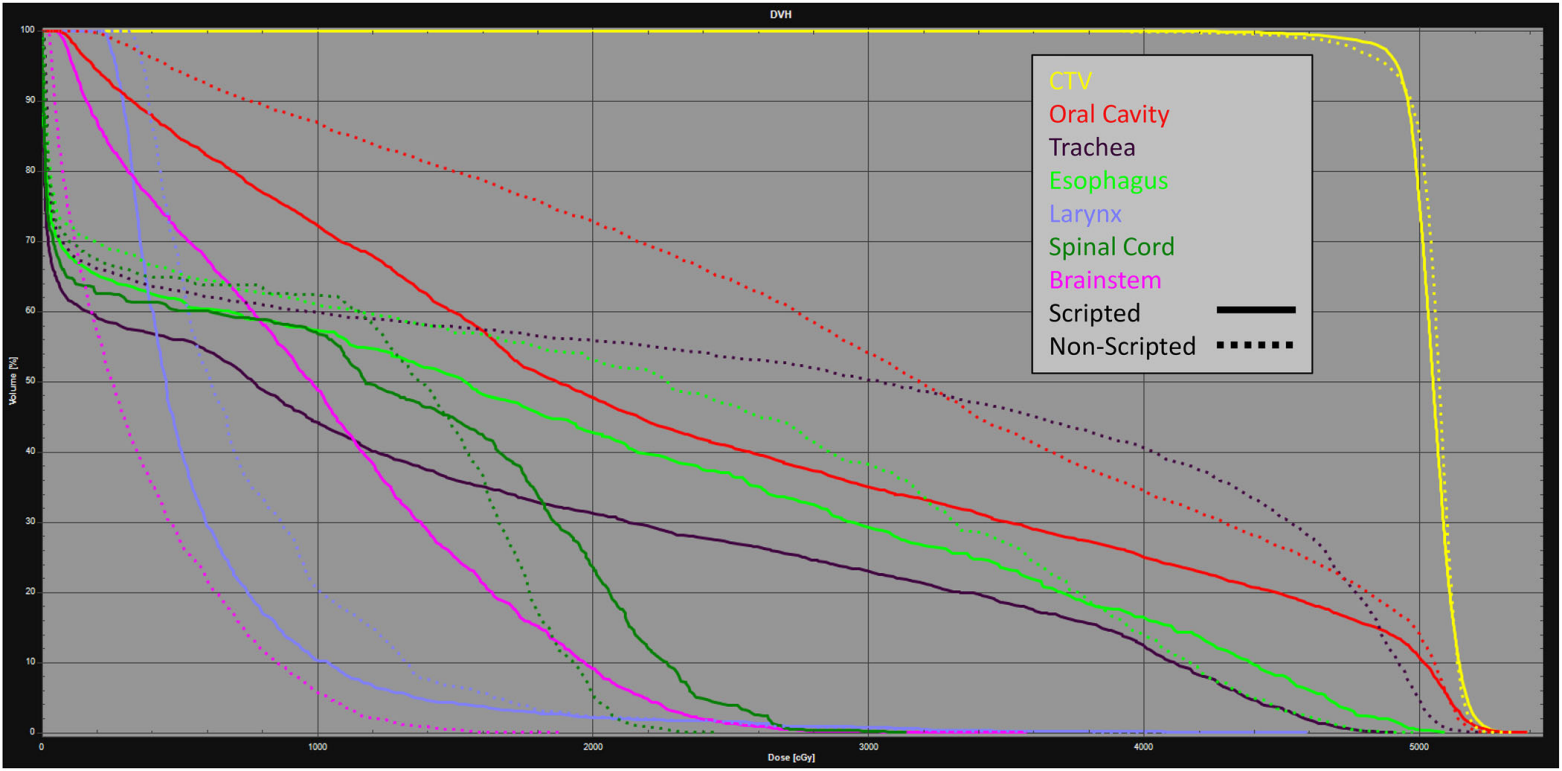
DVH comparison for an example patient, including the CTV (yellow), oral cavity (red), trachea (purple), esophagus (bright green), larynx (lilac), spinal cord (dark green), and brainstem (pink). Scripted plans are solid, non‐scripted plans are dashed

The average perceived effort for a scripted versus non‐scripted plan was 39.7 and 68.6, respectively (*p* < 0.05). Because this value is subjective to the planner, it is also interesting to evaluate each planner, rather than comparing each patient. Figure 4 demonstrates the comparison of perceived effort for each planner.

A single physicist performed the evaluation for each plan. For two of the non‐scripted plans, the planner was contacted during evaluation to correct an error. In one case, an incorrect avoid structure was created and applied. In the other case, the planner was too aggressive when sparing the spinal cord, and robustness failed for the target coverage. In both cases, the planners fixed the errors and the additional time to fix the mistake was added to the results.

**FIGURE 5:**
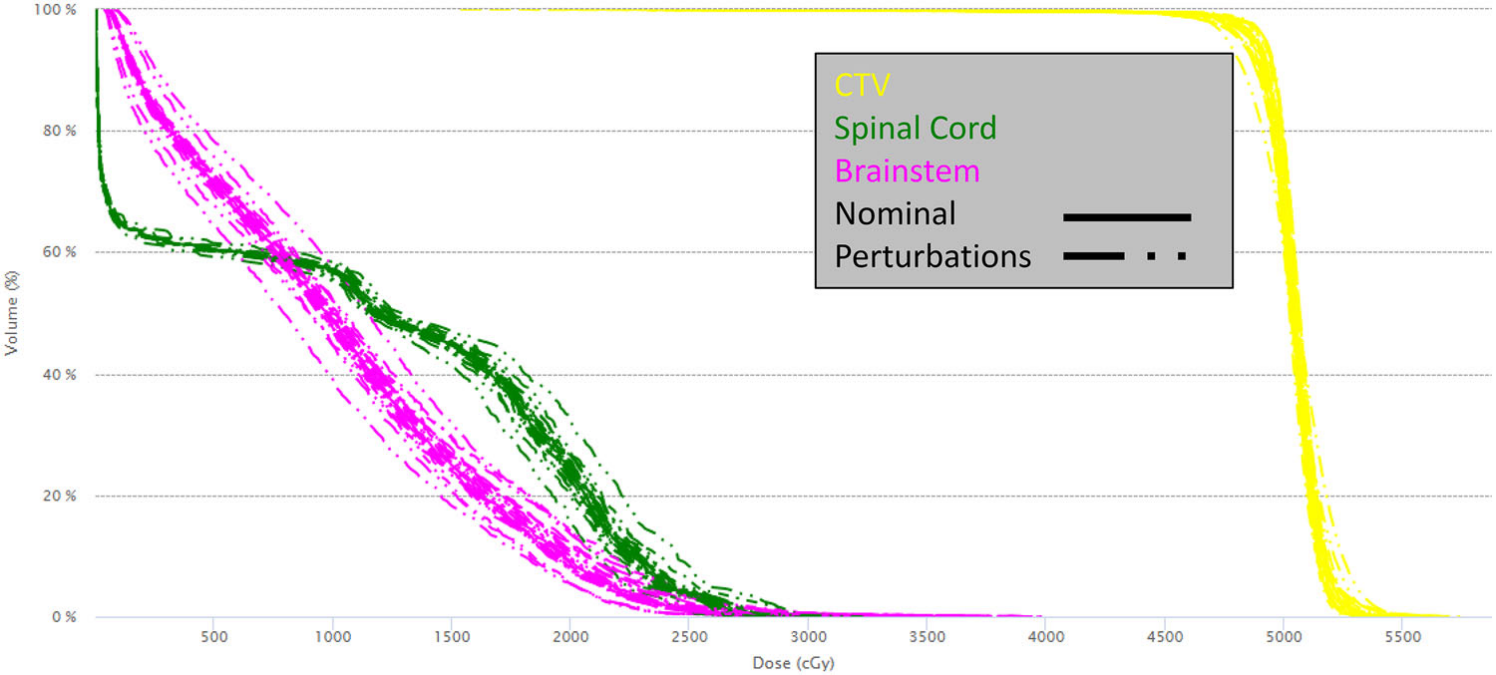
Summary of robust perturbations for an example patient, including shifts, range uncertainty, rotations, and independent beam shifts. CTV (yellow), spinal cord (green), brainstem (pink). Nominal plan is solid, perturbations are dashed

For the robust evaluation, 24 perturbations were calculated, including 3 mm patient shifts in +/− *X*, *Y*, and *Z* directions, +/− 3.5% range uncertainty, +/− 3° roll and yaw, and 3 mm shifts of both an anterior and a posterior beam independently in +/− *X*, *Y*, and *Z* directions. Robust evaluations confirmed that optimizing with lower robust settings than the evaluation criteria can still produce a robust PBS plan. There was no difference in robust target coverage between scripted and non‐scripted plans. The CTV50 *D*95% did not change by more than 3% on any perturbation, per our in‐house requirements for robust coverage. For the CTV50, on average, across all 24 perturbations, 86% of the perturbed DVH points were within 2% of the nominal DVH and 95% of the perturbed DVH points were within 5% of the nominal DVH for scripted plans. For non‐scripted plans, 88% were within 2% and 95% were within 3%. Figure 5 demonstrates a typical DVH band for the CTV50, spinal cord, and brainstem, including 24 perturbations and the nominal plan.

## 4 DISCUSSIONS

The purpose of this work was to validate a fully automated multi‐beam MFO proton planning script. The goals of the automation were to standardize the planning process and reduce time and effort for the treatment planner. Interestingly, automation did not significantly reduce planning time. However, we did show that automation improved nominal plan quality, based on OAR sparing, and perception of effort. The planning technique used for these patients is complex, requiring specific beam settings, avoidance structures, robust optimization settings, and planning objectives and constraints. These complexities can be difficult to remember and implement, even with good standard operating procedures. Automation can streamline these complex processes and perform most of the work, without user input, hence the reduction of perceived effort through automation. When evaluating the scripted plans, each plan passed initial plan evaluation and robust evaluation. Two of the non‐scripted plans had to be corrected, further proving the advantage of automating complex techniques. This planning process was designed and optimized to provide the best possible target coverage and robustness balanced with OAR sparing. If a user neglects an aspect of the process, for example, uses a wrong or different avoid structure or planning objective, the plan quality and robustness will likely suffer. These off‐script plans often fail the evaluation process and require re‐planning, thus more time and effort is wasted.

There have been concerns that applying the same standardized and automated beam angles and planning objectives to every patient can reduce plan quality. However, through the hard‐coded use of validated planning objectives, robust settings, and patient‐specific predictive OAR objectives, we have proven otherwise and saw an improvement in plan quality. Scripted plans had lower mean doses to most structures, but the max doses to the brainstem and spinal cord were lower in the non‐scripted plans. Based on the outcomes of this study, the automation script has been updated to include better predictions for max doses, rather than focusing only on mean doses. The max dose reduction is an important finding, but the scripted plan values were well below tolerance for the first phase of treatment for each of those structures and therefore the improvement in max dose was not necessarily clinically significant.

There are many tools available, both in treatment planning software packages and third‐party packages, that can offer automated treatment planning and predictive OAR sparing. These tools can be expensive and are not typically based on the processes and dosimetry unique to each clinic. Additionally, there are far fewer tools available for proton therapy than for photon treatments. With most treatment planning software offering scripting options, each user is now capable of developing automation based on their own workflows and proven techniques. This study has validated the use of a script written in‐house, without requiring expensive add‐on packages or tools, or even formal scripting training. Other proton centers can write and utilize similar scripts to automate their processes, thus reducing the time and effort required per treatment plan. Considering the impact of time‐to‐treatment for H&N patients, particularly in proton therapy, this type of automation can be instrumental in improving clinical outcomes and referrals for patients. Due to the small‐sample size, there was no statistical difference in the time reduction with automation, but there is a clinical difference. The extra time required for a non‐scripted was the additional time required to manually create avoidance structures and input beams, optimization objectives, and clinical goals. There are many demands on a planner in a busy proton clinic, with planning patients for initial plans, boosts, adapts, and, often, comparative plans. By reducing the impact of each plan on the planner, it can reduce stress and allow a planning department to better allocate resources that improves overall time‐to‐treatment. Treatment planners can focus on more patients or spend more time on high level plan improvements, rather than basic plan setup.

## 5 CONCLUSIONS

Automating a multi‐beam MFO treatment planning process produces a high‐quality plan with good target coverage and robustness, as demonstrated with H&N. Automation reduces the time required to create a plan and reduces the perceived effort of the treatment planner. Incorporating patient‐specific predictive OAR sparing into a standardized and automated workflow can improve plan quality by reducing the mean dose to several critical structures. Each of the individual steps in the scripted planning process, including creation and use of avoidance structures, patient‐specific predicted OAR sparing objectives, and robust target coverage objectives, can be performed manually to develop a good PBS plan. However, including these steps within automation enforces the use of the tools and standardizes a high level of plan quality across all patients and planners. These scripts were developed in‐house, using tools provided by the treatment planning system.

## CONFLICT OF INTEREST

The authors declare that there is no conflict of interest that could be perceived as prejudicing the impartiality of the research reported.

## AUTHOR CONTRIBUTIONS

Samantha Grace Hedrick wrote the script, designed the study, analyzed the data, and wrote the paper. Scott Petro, Bart Morris, and Alex Ward designed the functions of the script.

## Supporting information



Supporting Information
