# Supplementary material for: Validation of automated complex head and neck treatment planning with pencil beam scanning proton therapy
Source: J Appl Clin Med Phys. 2021 Dec 22;23(2):e13510. doi: 10.1002/acm2.13510 (PMC8833278; doi:10.1002/acm2.13510)
Supplement: Supplementary file 1 — Supporting Information [file ACM2-23-e13510-s001.docx]

**Supplementary Material**

**Description of the OAR prediction script**

Using multiple treatment sites, such as H&N, brain, and prostate, we found the typical dose fall-off around a target of our system. The script then uses this fall-off to simulate isodose rings around the target at every 10% of prescription dose. A graphic user interface (GUI) asks the user to indicate the target and, if planned robustly, the robust margins. If the user is planning to a PTV non-robustly, the target is assumed to the selected target. If the user is planning robustly to a CTV, the script will first add a uniform robust margin to the target and then create the simulated isodose rings based on that expanded target. The use of a uniform contour margin added for robustness is conservative and tends to be larger than the actual dose margin. These simulated isodose rings are unioned with each OAR of interest and the volume of OAR is calculated. It is then assumed that the relative volume of the OAR would receive the corresponding simulated dose. The mean dose is calculated by summing each 10% isodose intersection with the OAR in question and multiplying by 10% of the Rx dose. This summation gives the total dose to the OAR in question. That dose is then divided by the total volume of the OAR to give the average dose to the OAR. The script was validated to accurately predict isodose rings for 100% of prescription, 90%, 80%, 70%, and 60%. At lower isodoses, below 60%, the dose fall-off around the target is heavily dependent on beam angle due to entrance dose. However, in our case of H&N, where we are treating with beams spaced semi-evenly around the patient, the prediction is less susceptible to beam angle dependency and is valid to lower isodoses. It can accurately predict mean dose for the parotids, oral cavity, larynx, and trachea, which are typically peripheral to the target. It is not used for structures like the esophagus, which is typically in the middle of the target and not well predicted. Currently, the script cannot predict SIB dose distributions, but that work is in progress.

| **Table S1** |  |
| --- | --- |
| Planning objectives and constraints | |
| *Structure* | *Planning Objective* |
| CTV50 | Dmin = 50 CGE |
|  | V97% = 97%* |
| Parotid_L | Predicted mean |
| Parotid_R | Predicted mean |
| OralCavity | Predicted mean |
| Esophagus | Predicted mean |
| Larynx | Predicted mean |
| Trachea | Predicted mean |
| Lungs | Dose Fall Off (50 CGE to 25 CGE in 1cm) |
| PharynxConst | Dmax = 50.5 CGE |
| BrachialPlexus | Dmax = 50.5 CGE |
| Brainstem | Dmax = 35.0 CGE |
| SpinalCord | Dmax = 35.0 CGE |
| Skin | Dmax = 46.5 CGE |
| External | Dmax = 51.5 CGE |
|  | Dmax = 54.0 CGE* |
|  | Dose Fall Off (50 CGE to 25 CGE in 1cm) |
| PostNeck | Dmax = 20.0 CGE |
| SFO | Dmax = 27.5 CGE per anterior beam |
| * indicates robust optimization objective | |

| **Table S2** |  |  |
| --- | --- | --- |
| Composite OAR doses | | |
| *Structure* | *Scripted [CGE]* | *Non-Scripted [CGE]* |
| Parotid_L [mean±SD] | 28.20±6.96 | 32.25±7.14 |
| Parotid_R [mean±SD] | 22.78±11.8 | 27.86±11.97 |
| OralCavity [mean±SD] | 36.15±7.60 | 39.23±8.14 |
| Esophagus [mean±SD] | 10.35±5.75 | 12.46±5.41 |
| Larynx [mean±SD] | 38.19±24.11 | 44.73±22.56 |
| Trachea [mean±SD] | 19.18±5.25 | 26.78±7.90 |
| BrainStem [max±SD] | 24.81±11.15 | 19.89±8.41 |
| SpinalCord [max±SD] | 34.90±4.50 | 28.30±7.62 |
